# Harmonized Tubular Maximum Phosphate reabsorption per Glomerular Filtration Rate reporting in adults and children in Australia and New Zealand

**DOI:** 10.1093/jbmrpl/ziae140

**Published:** 2025-12-06

**Authors:** Cherie Chiang, Kay Weng Choy, Penelope Coates, Simon Carrivick, Christopher J Farrell, Roderick J Clifton-Bligh, Ee Mun Lim

**Affiliations:** Department of Medicine, University of Melbourne, Parkville, VIC 3050, Australia; Department of Endocrinology, Austin Health, Heidelberg, VIC 3084, Australia; Department of Pathology, Northern Health, Epping, VIC 3076, Australia; Department of Chemical Pathology, SA Pathology, Adelaide, SA 5000, Australia; Department of Biochemistry, Pathwest QEII Medical Centre, Nedlands, SA 6009 Australia; New South Wales Health Pathology, Liverpool Hospital, Liverpool, NSW 2170, Australia; Department of Endocrinology, Royal North Shore Hospital, St Leonards, NSW 2065, Australia; Department of Biochemistry, Pathwest QEII Medical Centre, Nedlands, SA 6009 Australia

**Keywords:** tubular maximum phosphate reabsorption per glomerular filtration rate, hypophosphatemia, osteomalacia, X-linked hypophosphatemia (XLH), online calculator

## Abstract

Tubular maximum phosphate reabsorption per glomerular filtration rate (TmP/GFR) is a key diagnostic test for renal phosphate wasting. However, there is a lack of international consensus regarding the reporting of age-related reference intervals. The TmP/GFR Harmonization Working Group, formed by The Australasian Association for Clinical Biochemistry (AACB) and Laboratory Medicine and The Australian and New Zealand Bone and Mineral Society (ANZBMS), aimed to evaluate analytical differences amongst commercial creatinine and phosphate assays, harmonize age-specific cut-offs and compare the ANZBMS TmP/GFR online calculator with existing products. A total of 11 360 results from The Royal College of Pathologists of Australasia Quality Assurance Programs were analyzed to assess creatinine and phosphate assay performance amongst 5 in vitro diagnostic (IVD) companies. Ortho-Clinical Diagnostics analyzers had a positive bias of up to 32% for serum phosphate and up to 29% for TmP/GFR. The other IVD companies produced comparable results and are suitable for harmonized reference intervals. To date, the AACB-ANZBMS TmP/GFR online calculator is the only validated Isotope Dilution Mass Spectrometry-creatinine aligned tool which caters for both pediatric and adult individuals, providing automatic interpretive comments to aid clinicians managing patients with hypophosphatemia.

## Graphical Abstract

**Figure:**
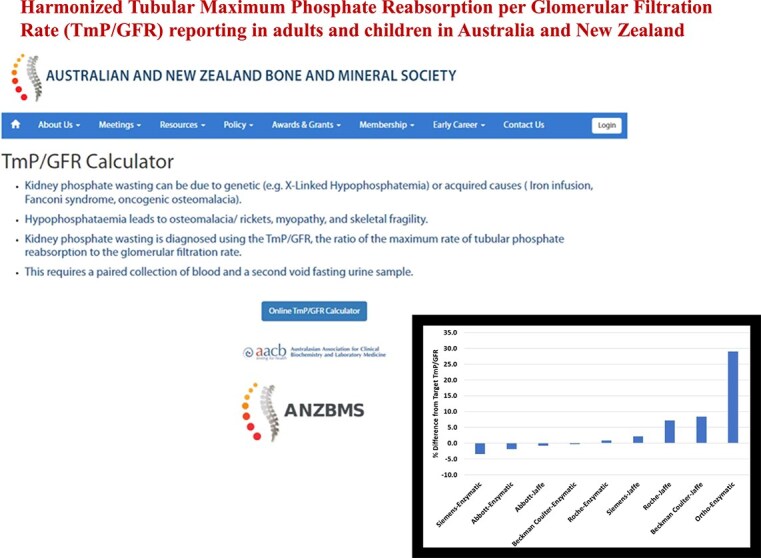

## Introduction

Tubular maximum phosphate reabsorption per glomerular filtration rate (TmP/GFR) is crucial for differentiating between gastrointestinal and renal phosphate wasting.^1-3^ The calculation involves a paired fasting blood and second void urine sample for creatinine and phosphate analysis, making it more accessible compared to FGF-23 and PHEX genetic testing. However, there is no international consensus on reporting age-related TmP/GFR reference intervals. Current online TmP/GFR calculators often use cut-offs derived from older Jaffe’s kinetic creatinine assays, which lack isotope-dilution mass spectrometry (IDMS) standardization. This standardization is critical for alignment of creatinine results from different manufacturers, accurate clinical interpretation, and estimated glomerular filtration rate (eGFR) predictions.^4^ There is also a lack of data on inter-assay biases which might impact on the final calculated TmP/GFR results, potentially leading to discordant findings between laboratories. Dubourg et al. retrospectively calculated age and sex-specific TmP/GFR reference intervals using IDMS standardized creatinine assays on 2 consecutive analyzers (Roche Modular and Abbott Architect) and recommended 20 separate age and sex specific reference intervals.^5^ However, no assessment was made on other analyzers such as the Ortho-Clinical Diagnostics Vitros analyzer which is popular in pediatric hospitals. The lack of consensus on age-specific cut-offs affects diagnosis and treatment. In particular, access to government-funded burosumab for X-linked hyphophosphatemia in Australia requires a low TmP/GFR according to age-specific reference intervals. The Australasian Association for Clinical Biochemistry (AACB), in collaboration with the Australian and New Zealand Bone and Mineral Society (ANZBMS), formed a working group to evaluate, harmonize, and verify age-related reference intervals and to provide an online TmP/GFR calculator for adult and children in Australia and New Zealand using IDMS-standardized creatinine assays.

## Materials and methods

### Creatinine and phosphate assay performance

The Royal College of Pathologists of Australasia Quality Assurance (RCPAQAP) General Serum and Urine Chemistry Programs were used to evaluate assay bias from 5 in vitro diagnostic (IVD) manufacturers of creatinine and phosphate assays: Abbott Diagnostics (Chicago, IL, United States), Beckman Coulter (Fullerton, CA, United States), Ortho-Clinical Diagnostics (Raritan, NJ, United States), Roche Diagnostics (Rotkreuz, Switzerland), and Siemens Healthcare Diagnostics (Erlangen, Germany). The 5 manufacturer medians were compared to the overall median and analytical performance specifications (APS) based on the Milan Consensus.^6^ All evaluated creatinine assays (Jaffe and enzymatic methods) were traceable to IDMS standards. One sample from the Serum and Urine Chemistry Program with concentrations compatible to an individual with renal phosphate wasting was used to calculate a target TmP/GFR, between method bias from the manufacturers were calculated using results submitted to the General Serum and Urine Chemistry Programs. The target TmP/GFR used the target creatinine and phosphate concentrations assigned to the material distributed to the laboratories.

### Harmonized TmP/GFR age-specific reference intervals and local data verification

The AACB-ANZBMS working group comprised of chemical pathologists and endocrinologists from 6 hospitals. The group assessed inter-assay bias and reached a consensus for age-related reference intervals.^7^ Published^8^ and local data from known renal phosphate wasting individuals and normal individuals at Pathwest QEII Medical Centre, SA Pathology, and Melbourne Health Pathology were verified against the harmonized IDMS-standardized creatinine assay-derived cut-offs.^5^ This project was approved by local ethics committee (MH HREC Number:2019.134).

### ANZBMS TmP/GFR online calculator comparison

TmP/GFR is calculated as follows^9^:

Tubular reabsorption of phosphorus (TRP) = 1 – [(serum creatinine x urine phosphate) / (serum phosphate x urine creatinine x 1000)].

If TRP ≤0.86, TmP/GFR = TRP x serum phosphate.

If TRP >0.86, TmP/GFR = (0.3 x TRP/ [1 – (0.8 x TRP)]) x serum phosphate.

(Serum creatinine value in μmol/L, serum phosphate in mmol/L, urine creatinine in mmol/L, and urine phosphate in mmol/L)

Search for available online calculators for TmP/GFR up to May 2024 was made and products were compared to assess whether they catered for pediatric individuals, had updated IDMS-creatinine aligned reference intervals, and the ability to generate automated interpretive comments.

### Statistical analysis

Results from the 5 manufacturers were presented as medians. Sensitivity and specificity analysis were performed using SPSS Statistic 28.0 (SPSS Inc., Chicago, IL, United States).

## Results

### Creatinine and phosphate assay performance

**Figure 1:**
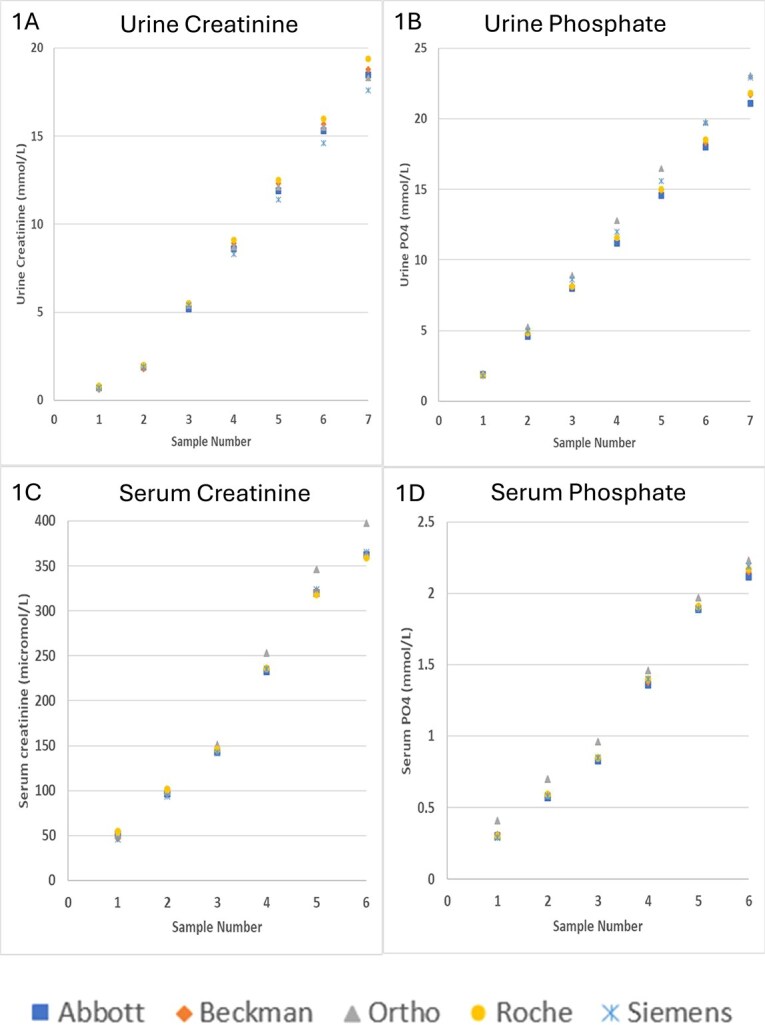
Manufacturer medians for quality assurance program samples. (A) Urine creatinine, (B) urine phosphate, (C) serum creatinine, (D) serum phosphate.

Twenty-two automated analyzers from 5 manufacturers performed creatinine and phosphate assays on both serum and urine samples in the 2024 RCPAQAP programs. In the urine program, 7 samples across a linear range were distributed to participating laboratories, resulting in 2625 creatinine results and 1435 phosphate results. In the serum program, 6 samples were distributed, yielding 3724 creatinine results and 3576 phosphate results. All urine creatinine and phosphate manufacturer medians were within APS targets, with biases ranging from −2.4% to 5.3% (APS target: ± 10%) and −5.3% to 10.4% (APS target: ± 15%), respectively. All serum creatinine manufacturer medians were within APS target of 8%, except for Ortho-Clinical Diagnostics, which showed a positive bias of 9.9% at a concentration of 362 μmol/L (Figure 1C).

All serum phosphate manufacturer medians were within APS of 8%, except for Ortho-Clinical Diagnostics, with a positive bias of up to 32% at concentrations between 0.31 and 0.85 mmol/L (Figure 1D).

**Figure 2:**
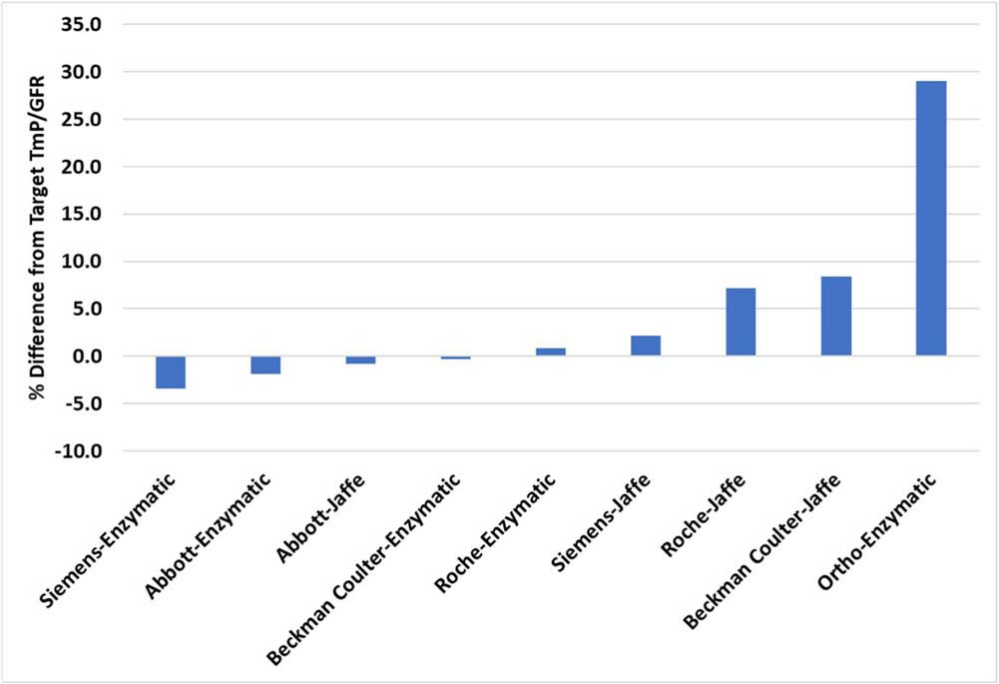
TmP/GFR bias calculated using a single quality assurance program material for serum and urine analyzed on assays from 5 manufacturers. Abbreviation: TmP/GFR, tubular maximum phosphate reabsorption per glomerular filtration rate.

Using a paired serum and urine chemistry RCPAQAP sample with a target TmP/GFR of 0.37 mmol/L (target serum creatinine = 51 μmol/L, serum PO4 = 0.58 mmol/L, urine creatinine = 5.3 mmol/L, urine PO4 = 21.8 mmol/L), the median results from 5 manufacturers were used to calculate analyzer-specific bias in TmP/GFR. The percentage difference compared to the target TmP/GFR varied between −3.4 and +29.1% (Figure 2). If Ortho-Clinical Diagnostics analyzers were excluded, the TmP/GFR bias varied between −3.4 and +8.4%.

### Harmonized TmP/GFR age-specific reference intervals and local data verification

**Figure 3:**
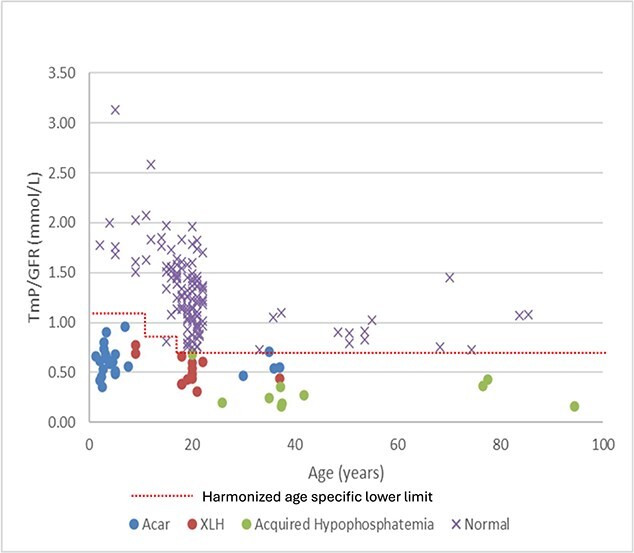
TmP/GFR in individuals with normal and renal phosphate wasting. Abbreviation: TmP/GFR, tubular maximum phosphate reabsorption per glomerular filtration rate.

After assessing the inter-method bias and least significant change for the assays, the working group condensed the age-specific reference intervals into 4 age-based categories, (Table 1) further stratification based on sex did not improve the sensitivity or specificity. Data from individuals with genetically confirmed XLH (*n* = 13), acquired hypophosphatemia (*n* = 11, 1 iron infusion, 5 hyperparathyroidism, 5 tubular dysfunction) and control individuals with normal renal phosphate handling (*n* = 121) were used to verify the new harmonized reference intervals. Published TmP/GFR data of known hereditary hypophosphatemia from Acar et al. (*n* = 22) were included.^8^ (Figure 3) The harmonized age-specific cut-offs diagnosed all XLH and acquired hypophosphatemia with 100% sensitivity and specificity. Among individuals with normal renal phosphate handling, one had a low TmP/GFR (15 yr-old with TmP/GFR = 0.81 mmol/L (reference interval, 0.87-1.69). One 35-yr-old participant from Acar et al.’s series had normal TmP/GFR of 0.71 (reference interval, 0.70-1.35). Using the harmonized cut-offs on the verification set resulted in a sensitivity of 97.8% (CI = 88.5-99.9%) and specificity of 99.2% (CI = 95.5-100%).

**Table 1:** AACB harmonized age specific reference intervals for TmP/GFR.

Age (years)	TmP/GFR (mmol/L)	TmP/GFR (mg/dL)
**0-<13**	1.11-1.70	3.44-5.27
**13-<16**	0.87-1.69	2.70-5.24
**16-<19**	0.71-1.61	2.20-4.99
**>19**	0.70-1.35	2.17-4.19

Abbreviations: AACB, Australasian Association for Clinical Biochemistry; TmP/GFR, tubular maximum phosphate reabsorption per glomerular filtration rate.

### ANZBMS TmP/GFR online calculator comparison

Four online TmP/GFR calculators were identified and compared to the ANZBMS calculator which was built using the verified harmonized age-specific reference intervals (Table 2). None of the calculators provided reference intervals based on IDMS-standardized creatinine assays. One calculator gave an error, one did not provide reference intervals for interpretation, and one did not provide age-specific reference intervals. The ANZBMS calculator was the only tool that allowed for age entry to generate a TmP/GFR result with interpretive comments.

**Table 2:** Comparison of ANZBMS and online calculators for TmP/GFR using dataset from a kidney phosphate wasting individual.^a^

Calculator	IDMS-creatinine aligned	Age enterable as a parameter	Calculated TmP/GFR (TRP)	Automated generated comment
**ANZBMS, Australia** **https://www.anzbms.org.au/tmpgfr/tmpgfr-calculator.asp**	Yes	Yes	0.77 (0.81)	Yes, “Subject’s age is 13 yr. TmP/GFR is low, consistent with kidney phosphate wasting.”
**Gesellschaft für pädiatrische Nephrologie, Germany** **https://gpn.de/service/tmp-gfr-calculator**	No	No	0.77 (0.81)	No, age-specific ranges provided in a table.
**Teveritt, United Kingdom** **http://www.teveritt.co.uk/calculations/renal_tubular_reabsorption_of_ph.htm**	No	No	0.77	No, no reference interval provided.
**Medicalc Scymed** **https://www.scymed.com/en/smnxps/pshpd274.htm**	No	No	(0.81)	No, only TRP provided with a single TRP cut off 0.82 used for all ages.
**Medical Professionals Reference** **https://www.empr.com/calculators/tubular-po4-reabsorption-calculator/**	No	No	Error, result could not be calculated	No, no reference interval provided.

^a^Age = 13 yr, plasma creatinine = 51 μmol/L (0.58 mg/dL), plasma PO4 = 0.95 mmol/L (2.94 mg/dL), urine creatinine 2.5 mmol/L (28.27 mg/dL), urine PO4 = 9 mmol/L (27.87 mg/dL). Normal TmP/GFR for age = 0.87-1.69.

Abbreviations: ANZBMS, Australian and New Zealand Bone and Mineral Society; IDMS, isotope-dilution mass spectrometry; TmP/GFR, tubular maximum phosphate reabsorption per glomerular filtration rate; TRP, tubular reabsorption of phosphorus.

## Discussion

Renal phosphate wasting can lead to rickets, osteomalacia, fractures, pain, and impaired quality of life.^10^ Whilst the causes are varied, TmP/GFR is a first-tier diagnostic test to confirm inappropriate kidney phosphate handling and exclude gastrointestinal phosphate loss using readily and rapidly available creatinine and phosphate assays.^1^^,^^2^ Unlike eGFR and creatinine clearance which are automatically calculated, few laboratories offer automated calculation of TmP/GFR despite availability of all required parameters within the laboratory information system. Due to the complexity of the calculation, clinicians often rely on web-based calculators to provide results and interpretations. We found that available calculators may provide conflicting results due to the lack of age-specific reference intervals or the use of intervals which were not IDMS-aligned. The original nomogram to assess renal threshold for phosphate handling by Walton et al. in 1975^11^ used creatinine and phosphate colorimetric assays prior to availability of international reference materials and the rigor of standardization. Furthermore, TmP/GFR reference values were established before the global wave of IDMS creatinine assay standardization, which enabled the use of eGFR across different manufacturers of creatinine assays.^4^ Dubourg et al. performed a single-cohort retrospective study consisting of 340 adults and 1711 children with normal renal function, plasma calcium, and phosphate to derive age-specific reference intervals using IDMS aligned creatinine assays from 2 manufacturers.^5^ Whether these reference intervals are compatible with other manufacturers of creatinine and phosphate assay is unknown.

The AACB TmP/GFR Harmonization Working Group evaluated inter-method bias via the RCPAQAP program and found a significant positive bias in low serum phosphate results from Ortho-Clinical Diagnostics analyzers, which translated to a 29% positive bias in TmP/GFR results calculated using creatinine and phosphate results from Ortho-Clinical Diagnostics instruments, popular amongst pediatric hospitals due to the small blood volume requirement. TmP/GFR results from the other 4 diagnostic companies did not reveal a significant bias, therefore harmonized reference intervals can be applied across these assays. Inherited renal phosphate wasting usually presents in childhood,^12^ when the normative TmP/GFR reference intervals are significantly higher than that in adulthood. From our verification studies using local and published data, there was a clear dichotomy between normal and abnormal renal phosphate handling in pediatric individuals. Therefore, it is reasonable for Ortho-Clinical Diagnostics users to use the ANZBMS TmP/GFR calculator, and to consider retesting urine and serum on an alternative instrument only if a normal TmP/GFR result was returned.

TmP/GFR declines sharply in both sexes during puberty before stabilizing in adulthood. The Working Group reviewed local reference intervals and harmonized them into 4 separate age-specific categories, which were verified using local and published data with high diagnostic accuracy. While we used a correction when TRP was above 86% regardless of age,^3^^,^^9^ some groups found that correction was not required in children.^13^ Dubourg et al. did not correct for participants below 19 yr of age due to concern that correction might overestimate TmP/GFR. Their study only extracted individuals with normal renal phosphate handling.^5^ In our verification study, all 17 of the hereditary renal phosphate wasting children from Acar’s series (age 1.3-7.5 yr old) and all 5 of the local XLH participants (age 9-19 yr old) had TRP less than 86% and correction was not required. Therefore, the universal use of TRP dependent correction did not alter the accuracy of the proposed harmonized reference ranges. Furthermore, all the available online TmP/GFR calculators correct for high TRP regardless of age. In fact, the ability to enter age as a parameter was not available in the online calculators we evaluated. At the time of publication, the ANZBMS TmP/GFR calculator was the only tool that enabled age-specific, IDMS-aligned reference intervals with automated interpretation based on the age entered. Future studies are required to verify the harmonized reference intervals in other populations. However, there is no known geographical or population difference for renal phosphate handling. In fact, adults and children manufacturer specific reference intervals for plasma and urine creatinine and phosphate are applicable globally. While this is a local population verification study, there is no physiological rationale for the TmP/GFR cut-off to differ in population outside of Australia and New Zealand unless they use a manufacturer not studied in this paper.

One limitation of our verification study is the use of a retrospective cohort due to the small number of participants with genetically proven hereditary renal phosphate wasting. The inter-assay bias was extrapolated from external quality assurance (EQA) programs, with uncertain commutability properties compared to true human serum and urine. However, positive bias for phosphate was also noted for the Ortho-Clinical Diagnostics group in the HAPPI kids study which evaluated 30 analytes across 5 diagnostic companies using blood from healthy children, consistent with our EQA findings.^14^ Our findings are applicable to the 5 manufacturers who provide creatinine and phosphate assays used in Australia and New Zealand. These manufacturers also provide most of the services in Europe and United States. However, we do not have the assay bias data for manufacturers such as Mindray and Hitachi which have clients in South East Asia, therefore our TmP/GFR calculator might not be suitable for these assays.

In conclusion, harmonized age-specific reference intervals are compatible with routine chemistry assays except for Ortho-Clinical Diagnostics analyzers. The ANZBMS TmP/GFR calculator (https://www.anzbms.org.au/tmpgfr/tmpgfr-calculator.asp) generates interpretive comments based on the individual’s age, thereby providing a useful aid for clinicians who manage adults and children with persistent hypophosphatemia.

## Data Availability

Data available on request.

## References

[1] Tebben PJ . Hypophosphatemia: a practical guide to evaluation and management. Endocr Pract. 2022;28(10):1091-1099. 10.1016/j.eprac.2022.07.005 35940468

[2] Haffner D, Emma F, Eastwood DM, et al. Clinical practice recommendations for the diagnosis and management of X-linked hypophosphataemia. Nat Rev Nephro*l*. 2019;15(7):435-455. 10.1038/s41581-019-0152-5 31068690 PMC7136170

[3] Kenny AP, Glen AC. Tests of phosphate reabsorption. Lancet (London, England*)*. 1973;2(7821):158. 10.1016/s0140-6736(73)93112-7 4124087

[4] Pottel H, Cavalier E, Björk J, et al. Standardization of serum creatinine is essential for accurate use of unbiased estimated GFR equations: evidence from three cohorts matched on renal function. Clin Kidney *J*. 2022;15(12):2258-2265. 10.1093/ckj/sfac182 36381377 PMC9664577

[5] Derain Dubourg L, Aurelle M, Chardon L, Flammier S, Lemoine S, Bacchetta J. Tubular phosphate handling: references from child to adulthood in the era of standardized serum creatinine. Nephrol Dial Transplant. 2022;37(11):2150-2156. 10.1093/ndt/gfab331 34850142

[6] Sandberg S, Fraser CG, Horvath AR, et al. Defining analytical performance specifications: consensus statement from the 1st strategic conference of the European Federation of Clinical Chemistry and Laboratory Medicine. Clin Chem Lab Me*d*. 2015;53(6):833-835. 10.1515/cclm-2015-0067 25719329

[7] Tate JR, Sikaris KA, Jones GR, et al. Harmonising adult and paediatric reference intervals in Australia and New Zealand: an evidence-based approach for establishing a first panel of chemistry analytes. Clin Biochem Rev. 2014;35(4):213-235. 25678727 PMC4310061

[8] Acar S, BinEssa HA, Demir K, et al. Clinical and genetic characteristics of 15 families with hereditary hypophosphatemia: novel mutations in PHEX and SLC34A3. PLoS On*e*. 2018;13(3):e0193388. 10.1371/journal.pone.0193388 29505567 PMC5837132

[9] Payne RB . Renal tubular reabsorption of phosphate (TmP/GFR): indications and interpretation. Ann Clin Bioche*m*. 1998;35(2):201-206. 10.1177/000456329803500203 9547891

[10] Cheung M, Rylands AJ, Williams A, Bailey K, Bubbear J. Patient-reported complications, symptoms, and experiences of living with X-linked hypophosphatemia across the life-course. J Endocr Soc. 2021;5(8):bvab070. 10.1210/jendso/bvab070 34258488 PMC8272533

[11] Walton RJ, Bijvoet OL. Nomogram for derivation of renal threshold phosphate concentration. Lancet. 1975;2(7929):309-310. 50513 10.1016/s0140-6736(75)92736-1

[12] Munns CF, Yoo HW, Jalaludin MY, et al. Asia-Pacific consensus recommendations on X-linked hypophosphatemia: diagnosis, multidisciplinary management, and transition from pediatric to adult care. JBMR Plu*s*. 2023;7(6):e10744. 10.1002/jbm4.10744 37283655 PMC10241092

[13] Brodehl J, Krause A, Hoyer PF. Assessment of maximal tubular phosphate reabsorption: comparison of direct measurement with the nomogram of Bijvoet. Pediatr Nephrol (Berlin, Germany*)*. 1988;2(2):183-189. 10.1007/BF00862587 3153009

[14] Hoq M, Matthews S, Karlaftis V, et al. Reference values for 30 common biochemistry analytes across 5 different analyzers in neonates and children 30 days to 18 years of age. Clin Che*m*. 2019;65(10):1317-1326. 10.1373/clinchem.2019.306431 31481458

